# Recurrent histone mutations in T‐cell acute lymphoblastic leukaemia

**DOI:** 10.1111/bjh.15155

**Published:** 2018-03-30

**Authors:** Grace Collord, Inigo Martincorena, Matthew D. Young, Letizia Foroni, Niccolo Bolli, Michael R. Stratton, George S. Vassiliou, Peter J. Campbell, Sam Behjati

**Affiliations:** ^1^ Wellcome Trust Sanger Institute Wellcome Trust Genome Campus Hinxton Cambridgeshire UK; ^2^ Department of Paediatrics University of Cambridge Cambridge UK; ^3^ Centre for Haematology Faculty of Medicine Imperial College London London UK; ^4^ Clinical Haematology Imperial College Healthcare NHS Trust London UK; ^5^ Department of Oncology and Haemato‐Oncology University of Milan Milan Italy; ^6^ Department of Oncology and Haematology Fondazione IRCCS Istituto Nazionale dei Tumori Milan Italy; ^7^ Department of Haematology University of Cambridge Cambridge UK

**Keywords:** acute leukaemia, cancer genetics, aetiology, haematological malignancy

Mutations affecting key modifiable histone type 3 (H3; Table SI) residues are frequent oncogenic events in certain solid tumours (Feinberg *et al*, 2016), and have also recently been implicated in a subset of acute myeloid leukaemia (AML) (Lehnertz *et al*, 2017). Here, we systematically reviewed the somatic mutations in >20 000 cancer specimens to identify tumours harbouring H3 mutations. In a subset of T‐cell acute lymphoblastic leukaemia (T‐ALL) we identified non‐methionine mutations of the key modifiable H3 residues, lysine (K) 27 and 36.

The starting point of our investigation was a search for H3 hotspot mutations in 1020 human cancer cell lines (Table SII). In two cell lines, both derived from T‐ALL, we found lysine‐to‐arginine mutations at H3K27 and H3K36 (Table 1). One of the cell lines, LOUCY, is derived from a *NOTCH1* wild‐type adult T‐ALL (Ben‐Bassat *et al*, 1990). The second, CML‐T1, was derived from the T‐lymphoblastic blast crisis of chronic myeloid leukaemia (Kuriyama *et al*, 1989). Ten further T‐ALL cell lines lacked coding H3 mutations (Table SIII). In solid tumours, H3K27 and H3K36 are typically mutated to methionine (Fig 1) (Feinberg *et al*, 2016). However, recent functional studies of H3 lysine‐to‐isoleucine mutations in AML demonstrate that the latter also dramatically alter global H3 methylation and acetylation patterns (Lehnertz *et al*, 2017). Therefore, we speculated that lysine‐to‐non‐methionine mutations may also be drivers of a subset of T‐ALL.

**Table 1:** Type 3 histone mutations in T cell leukaemia

Sample name	Sample type	Donor age (years)	Donor sex	H3 mutation
LOUCY	Cell line derived from ETP‐ALL	38	Female	*HIST1H3G* p.K36R
CML‐T1	Cell line derived from the acute T‐lympoblastic blast crisis of CML	36	Female	*H3F3A* p.K27R
SJTALL174	Primary ETP‐ALL specimen	Unknown (paediatric)	Unknown	*H3F3A* p.K36R
SJTALL080	Primary T‐ALL specimen	Unknown (paediatric)	Unknown	*H3F3A* p.K27R
PD2752a	Primary T‐ALL specimen	30	Male	*H3F3A* p.K27N

Out of 141 T cell leukaemia specimens screened (12 cell lines and 129 primary samples), 5 (3·5%) harboured a missense mutation at a modifiable lysine residues K27 or K36. CML, chronic myeloid leukaemia; ETP‐ALL, early T cell precursor acute lymphoblastic leukaemia; T‐ALL, T cell acute lymphoblastic leukaemia.

**Figure 1:**
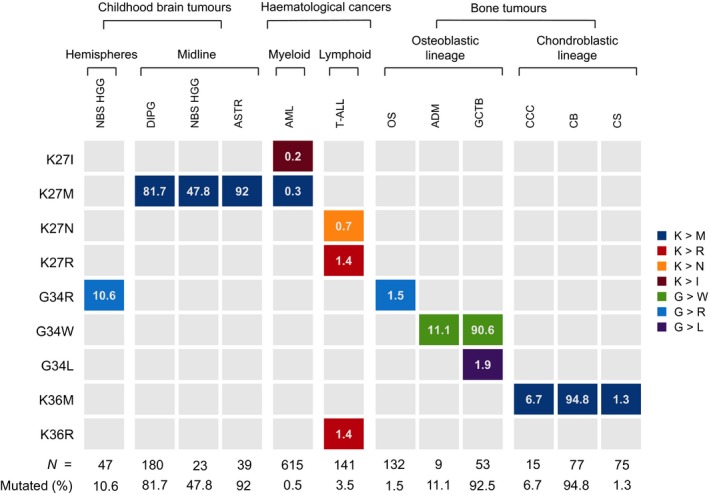
Prevalence and amino acid specificity of type 3 histone mutations in different cancer types. Columns indicate cancer types and rows show key histone type 3 regulatory residues. Tiles are coloured according to amino acid substitution. The percentage of each tumour type affected by the given class of histone mutation is indicated within the tiles and the overall prevalence of histone mutations is summarised at the bottom of each column. NBS HGG, non‐brain stem high grade glioma; DIPG, diffuse intrinsic pontine glioma; ASTR, astrocytoma; AML, acute myeloid leukaemia; T‐ALL, T cell acute lymphoblastic leukaemia; OS, osteosarcoma; ADM, adamantinoma; GCTB, giant cell tumour of bone; CCC, clear cell chondrosarcoma; CB, chondroblastoma; CS, chondrosarcoma.

We next searched for canonical H3 mutations in a published targeted sequencing study of 633 epigenetic regulator genes in >1000 childhood tumours encompassing 21 cancer subtypes (Huether *et al*, 2014). Amongst 91 T‐ALL specimens, there were two cases with canonical H3 mutations: *H3F3A* p.K27R and *H3F3A* p.K36R (Table I). Both mutations were clonal, with a variant allele fraction (VAF) of 38% and 55%, respectively. Among the 37 tumours with H3K mutations, lysine‐to‐arginine mutations were restricted to T‐ALL (*P* = 0·001502; Fisher's exact test).

We then extended our screen for H3 mutations to 18 704 tumours, encompassing >60 cancer types other than T‐ALL (Tables SIV and SV). This dataset comprised 8764 internally sequenced specimens and 9940 TCGA samples re‐analysed using an in‐house variant calling pipeline as previously described (Martincorena *et al*, 2017). We identified only one neomorphic H3 mutation in an acute leukaemia specimen: a previously reported *HIST1H3D* p.K27M mutation in an adult AML case (TCGA‐AB2927‐03) (Lehnertz *et al*, 2017).

Finally, we examined an additional T‐ALL cohort by capillary sequencing of recurrently mutated modifiable residues K27, G34, and K36 across four frequently mutated H3 genes (Tables SVI and SVII). The cohort comprised 38 T‐ALL cases described in detail previously (Maser *et al*, 2007). One specimen from a 30‐year‐old patient harboured a *H3F3A* p.K27N mutation (Figure S1). Interestingly, a *H3F3A* p.K27N mutation and a *H3F3A* p.K27T variant were previously identified in a T‐ALL RNA sequencing study (*n* = 31) (Atak *et al*, 2013). Collectively, our findings indicate that H3K27 and H3K36 mutations are recurrent in T‐ALL, a result we were able to reproduce across multiple different cohorts encompassing adult and paediatric cases.

This finding is congruent with the fact that mutations in *SETD2* and *EZH2*, methyltransferases that catalyse trimethylation (me3) of H3K36 and H3K27, respectively, are frequent T‐ALL drivers (Belver & Ferrando, 2016). Disruptive *SETD2* alterations occur in 7·8% of early T cell precursor acute lymphoblastic leukaemia (ETP‐ALL), an aggressive subtype with stem cell‐like features (Belver & Ferrando, 2016). Interestingly, both T‐ALL specimens with H3K36R mutations originated from ETP‐ALL (Table 1). Notably, mutually exclusive *SETD2* and H3K36/H3K34 mutations are reported in paediatric high grade glioma, where both result in reduced H3K36me3 mediated by *SETD2* (Feinberg *et al*, 2016). It is unclear whether a similar co‐mutation pattern exists in T‐ALL, as H3 genes have not been included in targeted sequencing panels used by the largest T‐ALL genomic studies (Belver & Ferrando, 2016).

The role of H3K27 modifications in T‐ALL pathogenesis is complex (Belver & Ferrando, 2016). It is plausible that mutations affecting this residue could impact the activity of several histone modifiers with established roles in T‐ALL pathogenesis. Loss‐of‐function mutations in *EZH2* or other core components of Polycomb repressive complex 2 (PRC2) are found in 42% of ETP‐ALL and 25% of T‐ALL overall (Belver & Ferrando, 2016). Impaired PRC2 catalytic activity in T‐ALL is associated with reduced H3K27me3, stemness and poor prognosis (Belver & Ferrando, 2016). *H3F3A* p.K27M mutations appear to act predominantly by blocking H3K27 di‐ and trimethylation and increasing H3K27 acetylation (Feinberg *et al*, 2016). Recent work demonstrates that H3K27I mutations in AML are associated with similar changes in H3 modification patterns (Lehnertz *et al*, 2017), suggesting that other non‐methionine mutations at modifiable H3 residues may influence the activity of PRC2 and other histone modifying enzymes. The lysine‐specific demethylases *JMJD3* and *UTX* are further important regulators of H3K27me3 distribution in T‐ALL (Belver & Ferrando, 2016), and it is conceivable that these enzymes may also be affected by H3K27 or H3K36 mutations.

A feature of H3 mutations in solid cancers is their exquisite tumour type specificity (Fig 1) (Feinberg *et al*, 2016). In this context, it is notable that 5/5 H3 mutations in T‐ALL identified by this survey are lysine‐to‐non‐methionine mutations, and 4/5 are lysine‐to‐arginine mutations. Out of the >20 000 tumour specimens screened for H3 variants, only two other samples harboured H3 lysine‐to‐arginine mutations, both at low VAF and in tumours with relatively high coding mutation burdens (TCGA‐BT‐A20Q‐01 and TCGA‐AN‐A0FW‐01). Hence, it is possible that lysine‐to‐arginine mutations confer particular selective advantage in the context of T cell leukaemogenesis.

In summary, ~3% of T‐ALL harbour non‐methionine variants in H3 genes at key modifiable lysine residues. Given the role of dysregulated H3K27/H3K36 modification in T‐ALL pathogenesis and the established prognostic significance of mutations in lysine‐specific histone modifiers (Belver & Ferrando, 2016), this finding warrants further investigation of the prevalence, clinical and functional significance of H3 mutations in T‐ALL. In light of the recent discovery of oncogenic H3K37 mutations in AML (Lehnertz *et al*, 2017), our findings suggest a broader role for histone mutations in acute leukaemias and clearly justify incorporation of H3 genes into haematological cancer sequencing panels.

## Authorship

S.B., M.R.S. and P.J.C. conceived and designed the study. G.C. and S.B. performed analysis with input from M.Y., I.M. and N.B. L.F. contributed materials. G.C. and S.B. wrote the manuscript with contributions from G.S.V. and P.J.C.

## Conflict of interest

The authors have no competing financial interests to declare.

## Supporting information


**Figure S1.** Histone 3 mutation in T‐ALL validation cohort.

**Table SI**. Type 3 histone genes.

**Table SII.** COSMIC version 81 cell lines screened for type 3 histone mutations.

**Table SIII.** T‐cell leukaemia lines screened for type 3 histone mutations.

**Table SIV.** Internal database screened for histone 3 mutations.

**Table SV.** TCGA cohort screened for histone 3 mutations.

**Table SVI.** Validation cohort of 38 primary human T‐ALL specimens screened by Sanger sequencing of histone 3 genes.

**Table SVII.** Primers used to Sanger sequence hotspot residues in histone 3 genes.
