# Supplementary material for: Recurrent histone mutations in T‐cell acute lymphoblastic leukaemia
Source: Br J Haematol. 2018 Mar 30;184(4):676–9. doi: 10.1111/bjh.15155 (PMC6766952; doi:10.1111/bjh.15155)
Supplement: Supplementary file 1 — Figure S1. Histone 3 mutation in T‐ALL validation cohort. [file BJH-184-676-s001.pdf]

Supplementary Figure 1

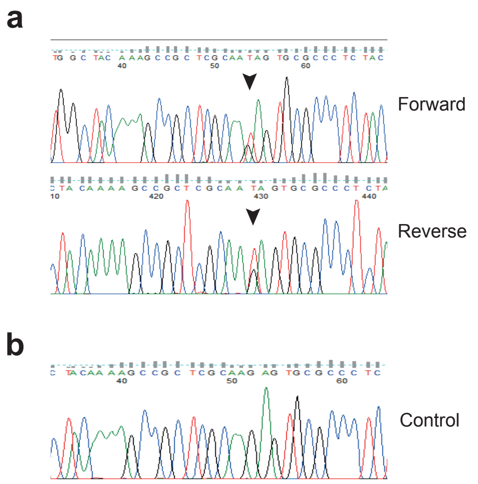

Supplementary Figure 1 | Histone 3 mutation in T-ALL validation cohort. (a) Bidirectional Sanger sequencing chromatograms of genomic T-ALL DNA showing a *H3F3A* p.K27N mutation (AAG>AAT). (b) Unidirectional trace from a control sample. T-ALL; T-cell acute lymphoblastic leukaemia.
