# Supplementary material for: Recurrent histone mutations in T‐cell acute lymphoblastic leukaemia
Source: Br J Haematol. 2018 Mar 30;184(4):676–9. doi: 10.1111/bjh.15155 (PMC6766952; doi:10.1111/bjh.15155)
Supplement: Supplementary file 2 — Table SI. Type 3 histone genes. [file BJH-184-676-s002.docx]

| **Supplementary Table 1. Type 3 Histone Genes** | |  |  |  |
| --- | --- | --- | --- | --- |
|  |  |  |  |  |
| **Approved symbol** | **Approved name** | **Previous symbols** | **Synonyms** | **Chromosome** |
| H3F3A | H3 histone family member 3A | H3F3 | H3.3A | 1q42.12 |
| H3F3AP1 | H3 histone family member 3A pseudogene 1 |  | HsT18159 | 15q15.1 |
| H3F3AP2 | H3 histone family member 3A pseudogene 2 |  | H3F3CP | 14q11.2 |
| H3F3B | H3 histone family member 3B |  | H3.3B | 17q25.1 |
| H3F3C | H3 histone family member 3C |  | H3.5 | 12p11.21 |
| HIST1H3A | histone cluster 1 H3 family member a | H3FA | H3/A | 6p22.2 |
| HIST1H3B | histone cluster 1 H3 family member b | H3FL | H3/l | 6p22.2 |
| HIST1H3C | histone cluster 1 H3 family member c | H3FC | H3/c, H3.1 | 6p22.2 |
| HIST1H3D | histone cluster 1 H3 family member d | H3FB | H3/b | 6p22.2 |
| HIST1H3E | histone cluster 1 H3 family member e | H3FD | H3/d, H3.1 | 6p22.2 |
| HIST1H3F | histone cluster 1 H3 family member f | H3FI | H3/i | 6p22.2 |
| HIST1H3G | histone cluster 1 H3 family member g | H3FH | H3/h | 6p22.2 |
| HIST1H3H | histone cluster 1 H3 family member h | H3FK | H3/k, H3F1K | 6p22.1 |
| HIST1H3I | histone cluster 1 H3 family member i | H3FF | H3/f, H3.f | 6p22.1 |
| HIST1H3J | histone cluster 1 H3 family member j | H3FJ | H3/j | 6p22.1 |
| HIST1H3PS1 | histone cluster 1 H3 pseudogene 1 |  | dJ45P21.6, H3F3AP1 | 6p22.2 |
| HIST2H3A | histone cluster 2 H3 family member a |  | H3/n, H3/o | 1q21.2 |
| HIST2H3C | histone cluster 2 H3 family member c | H3F2, H3FM | MGC9629, H3/m, H3, H3.2, H3/M | 1q21.2 |
| HIST2H3D | histone cluster 2 H3 family member d |  |  | 1q21.2 |
| HIST2H3PS2 | histone cluster 2 H3 pseudogene 2 |  | p06 | 1q21.1 |
| HIST3H3 | histone cluster 3 H3 | H3FT | H3t, H3/g, H3.4 | 1q42.13 |
