# Supplementary material for: Recurrent histone mutations in T‐cell acute lymphoblastic leukaemia
Source: Br J Haematol. 2018 Mar 30;184(4):676–9. doi: 10.1111/bjh.15155 (PMC6766952; doi:10.1111/bjh.15155)
Supplement: Supplementary file 3 — Table SII. COSMIC version 81 cell lines screened for type 3 histone mutations. [file BJH-184-676-s003.docx]

| **Supplementary Table 2. COSMIC version 81 cell lines screened for type 3 histone mutations** | | | |
| --- | --- | --- | --- |
|  |  |  |  |
| Primary histology | Histology subtype | Number of cell lines per histology subtype | Cell lines |
| carcinoma | NS | 168 | KMRC-1, CW-2, SUIT-2, KYSE-410, TCCSUP, MFE-319, EN, PCI-15A, OVKATE, KYSE-70, ChaGo-K-1, HT-115, HCT-15, HCT-116, SISO, NCC021, HSC-39, RKO, OVISE, FU97, ES-2, KYAE-1, KYSE-220, JIMT-1, TE-1, BICR31, C-33-A, HuCCT1, BICR78, HTC-C3, OV-90, BICR22, OVCAR-8, TE-10, H3118, JHH-1, CAL-85-1, KYSE-520, OACp4C, MDA-MB-453, IGROV-1, PL18, KMRC-20, NCC010, J82, 5637, TOV-21G, JHH-7, HCC1569, KYSE-50, Hs746T, KYSE-140, OCUB-M, TE-15, DSH1, SW780, Hs-578-T, KMH-2, SKG-IIIa, DoTc2-4510, TT2609-C02, PCI-6A, AU565, OSC-19, COLO-741, LB831-BLC, TE-8, MCF7, KP-3, JHU-011, OVTOKO, BT-20, YMB-1-E, TE-4, PCI-30, KP-1N, BICR10, CAL-51, ASH-3, IM-95, MDA-MB-361, SNU-398, EFM-192A, HCE-4, PL4, BFTC-909, IHH-4, KP-2, OVCAR-4, CAMA-1, BEN, NCI-H720, COLO-824, MDA-MB-436, SW156, Hs766T, KON, SAT, ML-1, HCC1500, HT-3, MRK-nu-1, OVMIU, JHH-4, HCC1428, JHU-029, A549, ESO26, MS751, PCI-38, UM-UC-3, MDA-MB-231, PA-TU-8902, NCI-H727, SCC90, Detroit562, NUGC-4, HeLa, JHU-022, PCI-4B, SKN-3, SW756, FU-OV-1, HDQ-P1, T-T, OCUM-1, RERF-GC-1B, OSC-20, OE21, MDA-MB-415, EC-GI-10, PE/CA-PJ15, HPAC, EVSA-T, MDA-MB-468, JHH-2, HCC202, RT4, TOV-112D, ESO51, OV-56, ME-180, A498, OAW-28, DAN-G, SK-GT-4, A427, JHH-6, VMRC-RCW, C-4-I, T84, SK-GT-2, HT-29, FLO-1, MDA-MB-330, PA-TU-8988T, T-24, OACM5-1, QGP-1, COLO-678, huH-1, SNU-182, PANC-04-03, LB647-SCLC, CAL-120, OVCAR-5, SU8686, PANC-02-03 |
| carcinoma | adenocarcinoma | 124 | HCC2998, NCI-H630, NCI-H1435, SNU-C5, NCI-H1793, SNU-407, LS-180, HUTU-80, SNU-81, SNU-175, GP5d, LNCaP-Clone-FGC, MKN45, NCI-H1993, NCI-H1568, CL-34, NCI-H1734, CaR-1, SNU-1040, AN3-CA, LC-2-ad, NCI-H2023, HCC-56, SW48, MKN28, DU-145, NCI-H1573, 201T, VMRC-LCD, EGI-1, NUGC-3, PC-14, HCC-827, KATOIII, A2780, SK-OV-3, AGS, SNU-283, HEC-1, VCaP, KM12, SW1463, LoVo, NCI-H660, SW1417, MDST8, SK-CO-1, EKVX, 23132-87, 22RV1, COLO-684, NCI-H2030, H3255, NCI-H1944, CCK-81, TGBC11TKB, SK-LU-1, LS-411N, NCI-SNU-16, NCI-H1781, SNU-C2B, HT55, MFE-296, NCI-H1975, TGBC1TKB, RF-48, C2BBe1, GEO, PC-3_[JPC-3], NCI-SNU-1, SW948, NCI-H1563, SNG-M, DIFI, SNU-61, COLO-205, HCC-44, LS-513, EMC-BAC-1, DOV13, GCIY, HOP-62, NCI-H716, LXF-289, LS-123, SNU-C1, SW1116, NCI-H1651, NCI-H508, CL-40, HCC-78, SW620, NCI-H2122, RCM-1, SK-HEP-1, Caov-3, HGC-27, ETK-1, RERF-LC-KJ, LIM1215, NCI-H1755, PC-3, EMC-BAC-2, LS-1034, MFE-280, SW837, Caov-4, MKN1, OE19, RERF-LC-MS, ABC-1, TMK-1, OVCAR-3, CL-11, SW403, NCI-SNU-5, COLO-320-HSR, KLE, COR-L105, NCI-N87, NCI-H3122, SW626, NCI-H747, OV-17R |
| carcinoma | small cell carcinoma | 67 | NCI-H1105, NCI-H847, NCI-H1341, NCI-H1155, HCC-33, NCI-H1048, RERF-LC-FM, NCI-H378, NCI-H2066, LU-135, LU-165, NCI-H526, NCI-H1836, COR-L279, NCI-H510A, NCI-H748, DMS-79, NCI-H446, NCI-H524, COR-L95, IST-SL2, NCI-H1417, NCI-H740, SW1271, MS-1, NCI-H2196, COR-L303, NCI-H64, NCI-H2227, NCI-H345, NCI-H146, SBC-1, NCI-H1436, NCI-H250, NCI-H841, NCI-H2081, NCI-H82, NCI-H1688, IST-SL1, DMS-273, NCI-H2141, NCI-H196, COR-L32, COLO-668, DMS-114, NCI-H1876, SHP-77, LU-134-A, COR-L88, LU-139, NCI-H211, SBC-3, NCI-H69, NCI-H1963, NCI-H1304, NCI-H2029, CPC-N, NCI-H1092, NCI-H2171, COR-L311, NCI-H187, DMS-53, NCI-H128, NCI-H1694, SBC-5, NCI-H209, TC-YIK |
| malignant melanoma | NS | 55 | SK-MEL-28, COLO-783, SH-4, CP50-MEL-B, Mewo, COLO-829, MEL-JUSO, IPC-298, M14, GAK, Hs940-T, UACC-257, MMAC-SF, MZ2-MEL, SK-MEL-2, 451Lu, LB373-MEL-D, MZ7-mel, SK-MEL-31, HMV-II, CP66-MEL, WM793B, COLO-792, CHL-1, WM-115, K2, A2058, IGR-1, RPMI-7951, Hs939-T, G-MEL, C32, VMRC-MELG, SK-MEL-5, SK-MEL-1, COLO-679, CP67-MEL, SK-MEL-30, WM35, LOXIMVI, MEL-HO, SK-MEL-3, RVH-421, WM278, SK-MEL-24, HT-144, A375, IGR-37, UACC-62, LB2518-MEL, G-361, COLO-800, IST-MEL1, A101D, WM1552C |
| carcinoma | squamous cell carcinoma | 53 | OMC-1, BHY, BB49-HNC, SAS, TE-5, SK-MES-1, KNS-62, HCC-15, HSC-3, EBC-1, SiHa, A431, CAL-33, SCC-4, LOU-NH91, NCI-H520, CAL-27, A388, KYSE-450, HARA, KYSE-180, HO-1-u-1, CAL-39, KYSE-150, TE-6, UDSCC2, HSC-4, SCC-9, Ca9-22, TE-12, HN, RPMI-2650, TE-11, KYSE-510, SCaBER, COLO-680N, FADU, RERF-LC-Sq1, LC-1-sq, Ca-Ski, KYSE-270, EPLC-272H, TE-9, HSC-2, SW962, SCC-15, LB771-HNC, KOSC-2, BB30-HNC, SW954, SCC-25, NCI-H1869, NCI-H226 |
| carcinoma | ductal carcinoma | 42 | ZR-75-30, BT-474, CFPAC-1, KP-4, HCC38, HPAF-II, DU-4475, CAL-148, PANC-03-27, HCC1954, YAPC, HCC1806, BT-549, MDA-MB-175-VII, HCC1395, HCC2218, AsPC-1, UACC-812, UACC-893, CAPAN-1, MIA-PaCa-2, MFM-223, HuP-T3, PANC-08-13, Capan-2, HCC1143, HuP-T4, SW1990, MZ1-PC, HCC1599, HCC1419, HCC2157, PANC-10-05, EFM-19, HCC1187, MDA-MB-157, BT-483, PSN1, HCC1937, T47D, HCC70, BxPC-3 |
| carcinoma | non small cell carcinoma | 39 | NCI-H2444, LU-65, NCI-H23, NCI-H1693, NCI-H2347, NCI-H1623, LK-2, NCI-H2135, NCI-H1770, NCI-H2342, NCI-H2110, NCI-H1648, NCI-H2172, NCI-H1355, NCI-H2170, HCC-366, NCI-H1437, NCI-H2009, NCI-H2085, NCI-H2126, COR-L23, NCI-H2405, NCI-H1838, NCI-H838, NCI-H1299, NCI-H661, SW1573, NCI-H2228, Calu-3, NCI-H647, CAL-12T, SW900, NCI-H2291, NCI-H2087, NCI-H522, Calu-6, NCI-H1792, NCI-H1650, NCI-H1395 |
| neuroblastoma | NS | 34 | MHH-NB-11, SIMA, NBsusSR, NH-12, KP-N-YS, SK-N-AS, NB7, NB14, TGW, SK-N-DZ, KP-N-YN, KP-N-RT-BM-1, NB13, SK-N-SH, NB(TU)1-10, LAN-6, NB5, NB17, NB10, NB69, ACN, NB12, CHP-134, CHP-212, SK-N-FI, GI-ME-N, MC-IXC, CHP-126, IMR-5, KELLY, GOTO, NB1, BE2-M17, NB6 |
| lymphoid neoplasm | B cell lymphoma unspecified | 29 | TUR, JSC-1, SU-DHL-6, Farage, SU-DHL-10, JEKO-1, HT, RL, VAL, RC-K8, BC-3, TK, MC116, CTB-1, SU-DHL-4, WSU-DLCL2, SLVL, OCI-LY-19, KARPAS-1106P, CRO-AP2, SC-1, SCC-3, SU-DHL-16, SU-DHL-5, BC-1, GRANTA-519, NU-DUL-1, JM1, OCI-LY7 |
| haematopoietic neoplasm | acute myeloid leukaemia | 27 | HEL, P31-FUJ, KO52, THP-1, OCI-M1, GDM-1, OCI-AML5, NOMO-1, ME-1, SIG-M5, KMOE-2, MONO-MAC-6, NB-4, NKM-1, CESS, CMK, ML-2, QIMR-WIL, OCI-AML2, PL-21, MOLM-16, OCI-AML3, KASUMI-1, KG-1, HL-60, MOLM-13, MC-1010 |
| glioma | NS | 24 | GB-1, H4, YKG-1, KALS-1, 8-MG-BA, KNS-81-FD, GAMG, SF295, LN-18, T98G, KNS-42, 42-MG-BA, U251, SF539, LN-229, NMC-G1, LNZTA3WT4, A172, SNB75, SF268, D-542MG, U-118-MG, U-87-MG, Hs683 |
| Ewings sarcoma-peripheral primitive neuroectodermal tumour | NS | 23 | EW-12, ES3, EW-11, ES1, CADO-ES1, SK-PN-DW, EW-24, MHH-ES-1, EW-22, EW-13, SK-ES-1, ES4, EW-3, EW-18, ES6, ES8, ES7, ES5, EW-16, EW-7, TC-71, A673, EW-1 |
| mesothelioma | NS | 20 | H2804, H513, H2595, NCI-H2452, H2722, H2461, H2369, H2810, IST-MES1, NCI-H28, MPP-89, H2731, H2869, H2818, H2373, H290, H2803, H2591, H2795, NCI-H2052 |
| lymphoid neoplasm | plasma cell myeloma | 19 | AMO-1, CTV-1, KMS-12-BM, MC-CAR, MM1S, SK-MM-2, RPMI-8226, KMS-11, NCI-H929, OPM-2, EJM, IM-9, MOLP-8, U-266, JJN-3, KARPAS-620, LP-1, ARH-77, L-363 |
| carcinoma | clear cell renal cell carcinoma | 14 | HA7-RCC, RCC-FG2, CAKI-1, 769-P, RCC-AB, LB996-RCC, RCC-ER, 786-0, RCC-JF, BB65-RCC, RCC-JW, RCC-MF, LB1047-RCC, LB2241-RCC |
| lymphoid neoplasm | acute lymphoblastic B cell leukaemia | 14 | REH, NALM-6, KOPN-8, RCH-ACV, MHH-PREB-1, MHH-CALL-2, U-698-M, BALL-1, MN-60, ROS-50, SUP-B8, KARPAS-231, SUP-B15, MHH-CALL-4 |
| lymphoid neoplasm | Burkitt lymphoma | 14 | Daudi, BL-70, NAMALWA, JiyoyeP-2003, DG-75, P32-ISH, BL-41, EB-3, Raji, EB2, Ramos-2G6-4C10, CA46, ST486, GA-10 |
| glioma | astrocytoma | 13 | D-392MG, SW1088, D-336MG, SK-MG-1, SF126, MOG-G-UVW, MOG-G-CCM, D-263MG, D-502MG, D-566MG, D-423MG, D-247MG, D-245MG |
| glioma | astrocytoma Grade IV | 11 | CAS-1, DBTRG-05MG, KS-1, Becker, M059J, AM-38, YH-13, DK-MG, CCF-STTG1, GMS-10, LN-405 |
| lymphoid neoplasm | acute lymphoblastic leukaemia | 11 | MOLT-4, KARPAS-45, GR-ST, BE-13, P30-OHK, KE-37, CCRF-CEM, LC4-1, HAL-01, 697, ALL-PO |
| lymphoid neoplasm | acute lymphoblastic T cell leukaemia | 11 | PF-382, SUP-T1, MOLT-13, MOLT-16, DND-41, P12-ICHIKAWA, ATN-1, TALL-1, RPMI-8402, ALL-SIL, LOUCY |
| carcinoma | renal cell carcinoma | 10 | TK10, OS-RC-2, RXF393, A704, SN12C, VMRC-RCZ, CAL-54, RCC10RGB, U031, ACHN |
| carcinoma | transitional cell carcinoma | 10 | 639-V, BFTC-905, CAL-29, VM-CUB-1, 647-V, SW1710, KU-19-19, HT-1197, HT-1376, RT-112 |
| carcinoma | large cell carcinoma | 9 | NCI-H1581, NCI-H650, LCLC-103H, NCI-H1915, IA-LM, NCI-H460, NCI-H810, LCLC-97TM1, HOP-92 |
| lymphoid neoplasm | Hodgkin lymphoma | 9 | RPMI-6666, L-428, SUP-HD1, KM-H2, L-1236, HD-MY-Z, L-540, HDLM-2, Hs-445 |
| osteosarcoma | NS | 9 | HOS, HuO9, U-2-OS, NOS-1, G-292_Clone_A141B1, MG-63, NY, HuO-3N1, Saos-2 |
| carcinoma | hepatocellular carcinoma | 8 | SNU-387, SNU-423, HUH-6-clone5, HuH-7, C3A, HLE, SNU-449, SNU-475 |
| carcinoma | serous carcinoma | 8 | EFO-21, PEO1, JHOS-4, OVCA433, OVCA420, JHOS-3, JHOS-2, Hey |
| rhabdomyosarcoma | NS | 6 | TE-441-T, SJRH30, KYM-1, RH-41, RH-1, A204 |
| chondrosarcoma | NS | 5 | CHSA0011, CHSA0108, CAL-78, CHSA8926, H-EMC-SS |
| haematopoietic neoplasm | blast phase chronic myeloid leukaemia | 5 | CML-T1, EM-2, LAMA-84, MEG-01, BV-173 |
| haematopoietic neoplasm | chronic myeloid leukaemia | 5 | RPMI-8866, KCL-22, JURL-MK1, K-562, KU812 |
| lymphoid neoplasm | diffuse large B cell lymphoma | 5 | A3-KAW, A4-Fuk, KARPAS-422, DB, DOHH-2 |
| lymphoid neoplasm | NS | 5 | NK-92MI, Jurkat, WIL2-NS, DEL, SR |
| NS | NS | 5 | SU-DHL-8, CRO-AP3, COR-L321, Hep_3B2_1-7, STS-0421 |
| sarcoma | NS | 5 | VA-ES-BJ, Sarc9371, SK-LMS-1, S-117, MES-SA |
| carcinoma | anaplastic carcinoma | 4 | 8505C, 8305C, CAL-62, BHT-101 |
| carcinoma | bronchioloalveolar adenocarcinoma | 4 | NCI-H358, NCI-H441, NCI-H322M, NCI-H1666 |
| carcinoma | follicular carcinoma | 4 | FTC-133, RO82-W-1, K5, CGTH-W-1 |
| glioma | astrocytoma Grade III | 4 | KINGS-1, no-11, SW1783, no-10 |
| lymphoid neoplasm | hairy cell leukaemia | 4 | MLMA, HC-1, Mo-T, BONNA-12 |
| carcinoma | mixed adenosquamous carcinoma | 3 | RL95-2, NCI-H596, NCI-H1703 |
| carcinoma | mucoepidermoid carcinoma | 3 | NCI-H292, HO-1-N-1, A253 |
| carcinoma | undifferentiated carcinoma | 3 | TGBC24TKB, TYK-nu, KURAMOCHI |
| fibrosarcoma | NS | 3 | SW684, Hs633T, HT-1080 |
| haematopoietic neoplasm | acute leukaemia of ambiguous lineage | 3 | KY821, MY-M12, MV-4-11 |
| lymphoid neoplasm | anaplastic large cell lymphoma | 3 | SU-DHL-1, SUP-M2, KARPAS-299 |
| lymphoid neoplasm | chronic lymphocytic leukaemia-small lymphocytic lymphoma | 3 | JVM-3, EHEB, JVM-2 |
| primitive neuroectodermal tumour-medulloblastoma | NS | 3 | ONS-76, D-283MED, PFSK-1 |
| carcinoid-endocrine tumour | NS | 2 | NCI-H835, UMC-11 |
| carcinoma | Merkel cell carcinoma | 2 | MCC13, MCC26 |
| carcinoma | mixed carcinoma | 2 | OV-7, UWB1_289 |
| carcinoma | mucinous carcinoma | 2 | EFO-27, OAW-42 |
| carcinoma | small cell adenocarcinoma | 2 | ECC10, ECC12 |
| choriocarcinoma | NS | 2 | JAR, JEG-3 |
| germ cell tumour | embryonal carcinoma | 2 | NTERA-2_cl_D1, NEC8 |
| leiomyosarcoma | NS | 2 | SK-UT-1, SKN |
| malignant fibrous histiocytoma-pleomorphic sarcoma | NS | 2 | MFH-ino, GCT |
| other | neoplasm | 2 | CS1, RKN |
| rhabdomyosarcoma | embryonal | 2 | RH-18, RD |
| adnexal tumour | other | 1 | DJM-1 |
| adrenal cortical carcinoma | NS | 1 | SW13 |
| Askins tumour | NS | 1 | TASK1 |
| carcinoma | carcinosarcoma-malignant mesodermal mixed tumour | 1 | ESS-1 |
| carcinoma | clear cell carcinoma | 1 | RMG-I |
| carcinoma | endometrioid carcinoma | 1 | OVK-18 |
| carcinoma | giant cell carcinoma | 1 | LU-99A |
| carcinoma | medullary carcinoma | 1 | TT |
| carcinoma | papillary carcinoma | 1 | BCPAP |
| carcinoma | serous micropapillary carcinoma | 1 | OC-314 |
| carcinoma | tubular adenocarcinoma | 1 | MKN7 |
| chordoma | NS | 1 | U-CH2 |
| germ cell tumour | mixed | 1 | NCCIT |
| germ cell tumour | teratoma | 1 | PA-1 |
| glioma | gliosarcoma | 1 | GI-1 |
| haematopoietic neoplasm | acute leukaemia | 1 | RS4-11 |
| haematopoietic neoplasm | acute leukaemic transformation of essential thrombocythaemia | 1 | Set2 |
| haematopoietic neoplasm | chronic eosinophilic leukaemia-hypereosinophilic syndrome | 1 | EoL-1-cell |
| haematopoietic neoplasm | myelodysplastic syndrome | 1 | SKM-1 |
| hyperplasia | NS | 1 | BPH-1 |
| leiomyoblastoma | NS | 1 | G-402 |
| liposarcoma | NS | 1 | SW872 |
| lymphoid neoplasm | adult T cell lymphoma-leukaemia | 1 | HH |
| lymphoid neoplasm | follicular lymphoma | 1 | WSU-NHL |
| lymphoid neoplasm | mycosis fungoides-Sezary syndrome | 1 | H9 |
| lymphoid neoplasm | NK-T cell lymphoma | 1 | YT |
| mesothelioma | biphasic | 1 | MSTO-211H |
| osteosarcoma | osteoblastic | 1 | CAL-72 |
| other | choriocarcinoma | 1 | SCH |
| other | dysplasia | 1 | DOK |
| other | metaplasia | 1 | OE33 |
| other | multipotential sarcoma | 1 | SJSA-1 |
| other | normal | 1 | PWR-1E |
| primitive neuroectodermal tumour-medulloblastoma | desmoplastic | 1 | Daoy |
| rhabdoid tumour | NS | 1 | G-401 |
| sex cord-stromal tumour | granulosa cell tumour | 1 | KGN |
| synovial sarcoma | NS | 1 | SW982 |
| Wilms tumour | NS | 1 | SK-NEP-1 |
