# Supplementary material for: Recurrent histone mutations in T‐cell acute lymphoblastic leukaemia
Source: Br J Haematol. 2018 Mar 30;184(4):676–9. doi: 10.1111/bjh.15155 (PMC6766952; doi:10.1111/bjh.15155)
Supplement: Supplementary file 4 — Table SIII. T‐cell leukaemia lines screened for type 3 histone mutations. [file BJH-184-676-s004.docx]

| **Supplementary Table 3. T-cell leukaemia lines screened for type 3 histone mutations** | | |  |
| --- | --- | --- | --- |
|  |  |  |  |
| Sample name | Primary histology | Histology subtype 1 | Type 3 histone mutation |
| PF-382 | lymphoid neoplasm | acute lymphoblastic T cell leukaemia |  |
| SUP-T1 | lymphoid neoplasm | acute lymphoblastic T cell leukaemia |  |
| CML-T1 | haematopoietic neoplasm | T-lymphoblastic blast phase chronic myeloid leukaemia | H3F3A p.K27R |
| MOLT-13 | lymphoid neoplasm | acute lymphoblastic T cell leukaemia |  |
| MOLT-16 | lymphoid neoplasm | acute lymphoblastic T cell leukaemia |  |
| DND-41 | lymphoid neoplasm | acute lymphoblastic T cell leukaemia |  |
| P12-ICHIKAWA | lymphoid neoplasm | acute lymphoblastic T cell leukaemia |  |
| ATN-1 | lymphoid neoplasm | acute lymphoblastic T cell leukaemia |  |
| TALL-1 | lymphoid neoplasm | acute lymphoblastic T cell leukaemia |  |
| RPMI-8402 | lymphoid neoplasm | acute lymphoblastic T cell leukaemia |  |
| ALL-SIL | lymphoid neoplasm | acute lymphoblastic T cell leukaemia |  |
| LOUCY | lymphoid neoplasm | acute lymphoblastic T cell leukaemia | HIST1H3G p.K36R |
