# Supplementary material for: Recurrent histone mutations in T‐cell acute lymphoblastic leukaemia
Source: Br J Haematol. 2018 Mar 30;184(4):676–9. doi: 10.1111/bjh.15155 (PMC6766952; doi:10.1111/bjh.15155)
Supplement: Supplementary file 5 — Table SIV. Internal database screened for histone 3 mutations. [file BJH-184-676-s005.docx]

| **Supplementary Table 4. Internal database screened for histone 3 mutations** | |
| --- | --- |
|  |  |
| **Cancer type** | **Number of samples** |
| Acute myeloid luekemia | 45 |
| Adenoid cystic carcinoma | 125 |
| Angiosarcoma | 36 |
| B-cell acute lymphoblastic leukemia | 234 |
| Bone and soft tissue tumours | 33 |
| Breast carcinoma | 2120 |
| Cholangiocarcinoma | 18 |
| Chondrosarcoma | 104 |
| Chordoma | 89 |
| CNS tumors | 40 |
| Colorectal carcinoma | 586 |
| Craniopharyngioma | 10 |
| Dendritic cell neoplasms | 14 |
| Diffuse large B cell lymphoma | 78 |
| Ependymoma | 23 |
| Ewing sarcoma | 58 |
| Gastric cancers | 28 |
| Head and neck tumors | 6 |
| Hepatocellular carcinoma | 41 |
| Kaposi sarcoma | 19 |
| Lung adenocarcinoma | 130 |
| Melanoma | 245 |
| Meningioma | 86 |
| Mesothelioma | 255 |
| Mixed lineage luekaemia | 11 |
| Multiple myeloma | 482 |
| Other myeloid malignancies | 1384 |
| Myeloproliferative disorders | 438 |
| Osteosarcoma | 344 |
| Other | 62 |
| Pancreatic | 46 |
| Pituitary adenoma | 20 |
| Prostate | 603 |
| Renal | 686 |
| Sarcoma | 24 |
| Other skin cancers | 143 |
| Unclassifiable sarcoma | 98 |
| **Total** | **8764** |
