# Supplementary material for: Recurrent histone mutations in T‐cell acute lymphoblastic leukaemia
Source: Br J Haematol. 2018 Mar 30;184(4):676–9. doi: 10.1111/bjh.15155 (PMC6766952; doi:10.1111/bjh.15155)
Supplement: Supplementary file 6 — Table SV. TCGA cohort screened for histone 3 mutations. [file BJH-184-676-s006.docx]

| **Supplementary Table 5. TCGA cohort screened for histone 3 mutations** | |  |
| --- | --- | --- |
|  |  |  |
| **Cancer type** | **TCGA abbreviation** | **Number of samples** |
| Adrenocortical carcinoma | ACC | 92 |
| Bladder Urothelial Carcinoma | BLCA | 416 |
| Breast invasive carcinoma | BRCA | 821 |
| Cervical squamous cell carcinoma and endocervical adenocarcinoma | CESC | 307 |
| Colon adenocarcinoma | COAD | 398 |
| Esophageal carcinoma | ESCA | 185 |
| Glioblastoma multiforme | GBM | 404 |
| Head and Neck squamous cell carcinoma | HNSC | 512 |
| Kidney Chromophobe | KICH | 66 |
| Kidney renal clear cell carcinoma | KIRC | 382 |
| Kidney renal papillary cell carcinoma | KIRP | 289 |
| Acute Myeloid Leukemia | LAML | 159 |
| Brain Lower Grade Glioma | LGG | 533 |
| Liver hepatocellular carcinoma | LIHC | 363 |
| Lung adenocarcinoma | LUAD | 403 |
| Lung squamous cell carcinoma | LUSC | 502 |
| Mesothelioma | MESO | 81 |
| Ovarian serous cystadenocarcinoma | OV | 438 |
| Pancreatic adenocarcinoma | PAAD | 185 |
| Pheochromocytoma and Paraganglioma | PCPG | 184 |
| Prostate adenocarcinoma | PRAD | 503 |
| Rectum adenocarcinoma | READ | 157 |
| Sarcoma | SARC | 259 |
| Skin Cutaneous Melanoma | SKCM | 471 |
| Stomach adenocarcinoma | STAD | 441 |
| Testicular Germ Cell Tumors | TGCT | 156 |
| Thyroid carcinoma | THCA | 504 |
| Thymoma | THYM | 123 |
| Uterine Corpus Endometrial Carcinoma | UCEC | 549 |
| Uterine Carcinosarcoma | UCS | 57 |
| **Total** | | **9940** |
