# Supplementary material for: Recurrent histone mutations in T‐cell acute lymphoblastic leukaemia
Source: Br J Haematol. 2018 Mar 30;184(4):676–9. doi: 10.1111/bjh.15155 (PMC6766952; doi:10.1111/bjh.15155)
Supplement: Supplementary file 7 — Table SVI. Validation cohort of 38 primary human T‐ALL specimens screened by Sanger sequencing of histone 3 genes. [file BJH-184-676-s007.docx]

| **Supplementary Table 6. Validation cohort of 38 primary human T-ALL specimens screened by Sanger sequencing of histone 3 genes** | | | |
| --- | --- | --- | --- |
|  |  |  |  |
| Sample ID | Age | Sex | H3 mutation |
| PD2716a | 17 | F |  |
| PD2717a | 19 | M |  |
| PD2718a | 16 | M |  |
| PD2719a | 14 | M |  |
| PD2720a | 9 | M |  |
| PD2721a | 33 | M |  |
| PD2722a | 26 | F |  |
| PD2724a | 55 | M |  |
| PD2725a | 46 | M |  |
| PD2726a | 25 | M |  |
| PD2727a | 39 | M |  |
| PD2728a | 24 | M |  |
| PD2729a | 42 | M |  |
| PD2730a | 26 | F |  |
| PD2731a | 19 | M |  |
| PD2732a | 46 | F |  |
| PD2733a | 21 | M |  |
| PD2734a | 37 | F |  |
| PD2735a | 27 | M |  |
| PD2736a | 16 | M |  |
| PD2737a | 36 | M |  |
| PD2738a | 8 | M |  |
| PD2739a | 31 | M |  |
| PD2740a | 35 | M |  |
| PD2741a | 37 | M |  |
| PD2742a | 44 | M |  |
| PD2743a | 2 | M |  |
| PD2744a | 25 | M |  |
| PD2745a | 39 | F |  |
| PD2746a | 32 | M |  |
| PD2747a | 32 | M |  |
| PD2748a | 7 | M |  |
| PD2749a | 19 | M |  |
| PD2750a | 44 | M |  |
| PD2751a | 17 | M |  |
| PD2752a | 30 | M | H3F3A p.K27N |
| PD2753a | 15 | M |  |
| PD2754a | 17 | M |  |
