# Supplementary material for: Recurrent histone mutations in T‐cell acute lymphoblastic leukaemia
Source: Br J Haematol. 2018 Mar 30;184(4):676–9. doi: 10.1111/bjh.15155 (PMC6766952; doi:10.1111/bjh.15155)
Supplement: Supplementary file 8 — Table SVII. Primers used to Sanger sequence hotspot residues in histone 3 genes. [file BJH-184-676-s008.docx]

| **Supplementary Table 7. Primers used to Sanger sequence hotspot residues in histone 3 genes** | | |
| --- | --- | --- |
|  |  |  |
| PRIMER_NAME | OLIGO1 | OLIGO2 |
| H3F3B_K27_G34_K36_cc17_73775173 | AACGACGAATCTCTCGAAGC | CTTATCTTCGGGGCGTCTTT |
| HIST1H3C_K27_G34_K36_cc6_26045721 | CTCATTGCAAATGGCTCGTA | GCTCGGTGGACTTCTGGTAG |
| HIST1H3B_K27_G34_K36_cc6_26032206 | AAGCGAAGATCGGTCTTGAA | GTTTTGCCATGGCTCGTACT |
| H3F3A_K27_G34_K36_cc1_226252135 | CATGGCTCGTACAAAGCAGA | GCAAAAAGTTTTCCTGTTATCCA |
